# Complete Genome Sequences of Four Novel *Escherichia coli* Bacteriophages Belonging to New Phage Groups

**DOI:** 10.1128/genomeA.00741-15

**Published:** 2015-07-16

**Authors:** Alexander B. Carstens, Witold Kot, Lars H. Hansen

**Affiliations:** a Department of Environmental Science, Aarhus University, Roskilde, Denmark; b Department of Biology, Faculty of Science, University of Copenhagen, Copenhagen, Denmark

## Abstract

Here, we describe the sequencing and genome annotations of a set of four *Escherichia coli* bacteriophages (phages) belonging to newly discovered groups previously consisting of only a single phage and thus expand our knowledge of these phage groups.

## GENOME ANNOUNCEMENT

Since Félix d’Hérelle’s discovery of the bacteriophage in 1917 (1), much effort has been put into understanding phages and their diversity (2, 3). This is especially true for phages of classic laboratory strains of *Escherichia coli* (2). Nonetheless, even with almost a hundred years of studies, the diversity of *E. coli* phages is not fully explored (3). Here, we present the complete sequences of four novel *E. coli* phages belonging to two newly discovered bacteriophage groups. This will extend our knowledge of the sequence diversity of *E. coli* phages.

The four phages were isolated from animal fecal samples on *E. coli* strain MG1655. The DNA isolation and library preparation was done using direct plaque sequencing as described before (4), with the minor modification that SDS was used in a final concentration of 0.1%. The library was sequenced using Illumina paired-end sequencing on the MiSeq platform as a part of a flowcell (250 cycles; Illumina). The total read yield was between 49,884 (CAjan) and 182,112 (JenP2) reads per library. Reads were trimmed and *de novo* assembled using CLC Genomic Workbench version 7.0.4 (CLC bio, Aarhus, Denmark). The assembly was cross-verified by SPAdes Genome Assembler version 3.1.0 (5).

Three regions with relatively low coverage in CAjan’s genome were confirmed by PCR, followed by Sanger sequencing reactions (Macrogen, Seoul, Korea). Automated annotation and open reading frame identification was performed using the RAST annotation server (6). This was followed by manual verification by screening all the predicted proteins against the NCBI protein database using BLASTp (7) and by using a Pfam domain search (8). CAjan, JenP1, JenP2 and JenK1 are all double-stranded DNA phages with genome sizes of 59,670, 607,454, 59,802, and 60,747 bp, respectively. The four phages have a G/C content between 43.2% (JenP2) and 44.7% (CAjan) with an average coverage in the assembly between 114.7-fold (CAjan) and 542.79-fold (JenP2). The genomes contain between 88 (JenK1) and 91 (CAjan) predicted genes, the majority of which could not be assigned a function. All four phages contain an operon with genes involved in queuosine biosynthesis, as has been seen in other phages (9). The function of queuosine is not completely understood but might be involved in the specificity of transcription and codon recognition (10) and might help the phage hijack the host transcription and translation machinery.

JenP1, JenP2, and JenK1 are closely related to enterobacterium phage 9g (GenBank accession number NC_024146), sharing 93%, 90%, and 96% sequence identity, respectively. Enterobacterium phage 9g was, until now, considered the sole representative of a novel phage genus (11). At the time of assembly CAjan had no close relatives, but a new *Escherichia* phage “Seurat” sharing 96% sequence identity with CAjan has recently been published (12). Seurat also belongs to a novel group of phages with no other close relatives. The four sequenced phages JenP1, JenP2, JenK1, and CAjan therefore expand the two phage groups and bring new insights to the diversity of *E. coli* phages.

### Nucleotide sequence accession numbers. 

Genome sequences have been deposited in GenBank under the accession numbers KP064094 (CAjan), KP719132 (JenP1), KP719133 (JenP2), and KP719134 (JenK1).
